# Determinants of the Morning-Evening Home Blood Pressure Difference in Treated Hypertensives: The HIBA-Home Study

**DOI:** 10.1155/2014/569259

**Published:** 2014-12-14

**Authors:** Lucas S. Aparicio, Jessica Barochiner, Paula E. Cuffaro, José Alfie, Marcelo A. Rada, Margarita S. Morales, Carlos R. Galarza, Marcos J. Marín, Gabriel D. Waisman

**Affiliations:** Hypertension Section, Internal Medicine Department, Hospital Italiano de Buenos Aires, Juan D. Perón 4190, C1181ACH Buenos Aires, Argentina

## Abstract

*Background*. The morning home blood pressure (BP) rise is a significant asymptomatic target organ damage predictor in hypertensives. Our aim was to evaluate determinants of home-based morning-evening difference (MEdiff) in Argentine patients. *Methods*. Treated hypertensive patients aged ≥18 years participated in a cross-sectional study, after performing home morning and evening BP measurement. MEdiff was morning minus evening home average results. Variables identified as relevant predictors were entered into a multivariable linear regression analysis model. *Results*. Three hundred sixty-seven medicated hypertensives were included. Mean age was 66.2 (14.5), BMI 28.1 (4.5), total cholesterol 4.89 (1.0) mmol/L, 65.9% women, 11.7% smokers, and 10.6% diabetics. Mean MEdiff was 1.1 (12.5) mmHg systolic and 2.3 (6.1) mmHg diastolic, respectively. Mean self-recorded BP was 131.5 (14.1) mmHg systolic and 73.8 (7.6) mmHg diastolic, respectively. Mean morning and evening home BPs were 133.1 (16.5) versus 132 (15.7) systolic and 75.8 (8.4) versus 73.5 (8.2) diastolic, respectively. Significant beta-coefficient values were found in systolic MEdiff for age and smoking and in diastolic MEdiff for age, smoking, total cholesterol, and calcium-channel blockers. *Conclusions*. In a cohort of Argentine medicated patients, older age, smoking, total cholesterol, and use of calcium channel blockers were independent determinants of home-based MEdiff.

## 1. Introduction

Home blood pressure monitoring (HBPM) is well accepted among patients [1] and has been recognized as superior to conventional blood pressure measurements in terms of refining hypertension diagnosis and control [2, 3]. It shows good reproducibility [4], ensured by blood pressure (BP) averages based on several readings performed over multiple days. In addition, it avoids white-coat effect, is free from observer dilution bias, and, compared to office BP, offers better prognostic value in predicting mortality and cardiovascular (CV) events [3, 5–7].

There are diurnal variations in HBPM averages, and current guidelines recommend the BP measurements to be performed at least in the morning and in the evening [8–12]. Up to this date these home-derived daytime BP fluctuations have been related to several factors such as male gender [13, 14], older age, use of antihypertensive medications, regular alcohol drinking [14–16], sleep apnea [14, 17], insomnia [18], pre- or postbathing time [16, 19], pre- or postprandial time [20], smoking and tobacco exposure [14, 21, 22], and past history of cardiovascular disease [14].

The diurnal HBPM rhythm may provide important prognostic information. For instance, studies have highlighted the stand-alone predictive ability of morning over evening BP for subclinical target organ damage [23, 24]. They have also shown that differences between morning and evening BP may be more pronounced in patients with hypertension and that there are discrepancies between ethnicities regarding whether or not the morning or evening BP is higher with HBPM: basically, the Northeastern Asian studies showed consistently higher morning BP [13, 15, 16, 25, 26] and the European studies showed higher evening home BP [14, 27, 28] or little difference in average [29]. Moreover, it has been shown that in individuals with morning hypertension, whose BP values in the evening were relatively lower than those in the morning, there is a higher risk of stroke [30]. On the other side of the diurnal spectrum, evening home BP also has a high prognostic significance [30, 31].

The home-based morning/evening BP difference (MEdiff) is a potentially useful index for the management of treated hypertensive patients, which is calculated as morning minus evening home BP. It is already known to be a significant predictor of left ventricular hypertrophy [32–34], independently of the average home BP levels.

In this study we attempted to clarify the determinants of home MEdiff in a standardized fashion in Argentine medicated patients referred to an institution for BP control.

## 2. Methods

### 2.1. Study Population

This was a cross-sectional study where medical records were reviewed retrospectively to extract data from hypertensive outpatients aged ≥18 years referred for an HBPM by their treating physicians to the Hypertension Section, Department of Internal Medicine,* Hospital Italiano de Buenos Aires*, Argentina (HIBA-Home study). All patients were receiving stable treatment for at least 4 weeks. The analyzed period was from April 2008 to April 2010.

We used the hospital's electronic medical database in order to obtain information on medical history, intake of medications, and smoking habits of each participant. Hypertension was classified as a conventional BP of at least 140 mmHg systolic or 90 mmHg diastolic and an average home BP of at least 135 mmHg systolic or 85 mmHg diastolic according to internationally accepted limits [8]. Smoking was defined as daily use of tobacco products. Body mass index (BMI) was body weight in kilograms divided by height in meters squared. Previous cardiovascular disease included ischemic heart disease, atrial fibrillation, or congestive heart failure. Previous cerebrovascular disorders included transient ischemic attack or stroke. Serum cholesterol and blood glucose were determined by automated enzymatic methods on venous blood samples within 6 months prior to HBPM. Diabetes mellitus was a self-reported diagnosis, a fasting blood glucose level of 7.0 mmol/L (126 mg/dL) in two measurements, or random ≥11.1 mmol/L (200 mg/dL), or use of antidiabetic drugs.

### 2.2. Home BP Measurements

According to a protocol already published by our group [35], patients recorded their morning, afternoon, and evening home BPs for 4 up to 5 consecutive days in a duplicated fashion with a 1-minute difference between readings, after 5 minutes of rest in the sitting position with an oscillometric device (Omron HEM-705CP, Omron Corp., Tokyo, Japan), previously validated according to the British Hypertension Society (BHS) standards [36], using the appropriate cuff size. They were instructed on how to perform these measurements by technician staff members and brought home a standardized leaflet with indications which reminded them to perform the measurements as follows: (1) on awakening before breakfast, before antihypertensive drug intake, and after morning micturition, (2) in the afternoon within a 4-hour period starting at 2 p.m., and (3) in the evening before supper, not before 8 p.m. In all cases, measurements were taken without talking or crossing their legs during the procedure and by using, whenever feasible, always the nondominant arm. For the purposes of this study, the morning and evening measurements were analyzed separately, and afternoon measurements were included in total BP averages.

### 2.3. Statistical Methods

All data are expressed as mean ± SD or percentage. For comparison of means and proportions, we applied *t* test and *χ* ^2^ statistics, respectively. We considered *P* < 0.05 as statistically significant. We constructed a model in which statistically significant variables associated with home MEdiff in the univariable analyses were entered into a linear regression multiadjusted analysis.

## 3. Results

### 3.1. Characteristics of Participants

In total, there were 376 patients included in the study, of which 9 were discarded due to lack of morning or evening measurements. Of the remaining 367 subjects who were finally included for analysis, 365 (99.5%) were Caucasians, 242 (65.9%) were women, and 43 (11.7%) were current smokers. All of the patients were taking one or more blood pressure-lowering drugs (mean ± SD: 2.2 ± 1.0): calcium-channel blockers (204, 55.6%), diuretics (136, 37.1%), ACE inhibitors (132, 36.0%), angiotensin receptor blockers (148, 40.3%), beta blockers (142, 38.7%), alpha blockers (15, 4.1%), and other drug groups (13, 3.5%). Age ranged from 25 to 91 years (mean ± SD: 66.2 ± 14.5). The number of total self-recorded BP measurements ranged from 16 to 28 (mean ± SD: 24.7 ± 2.9). Hyperlipidemia and diabetes mellitus were observed in 71.1% and 10.6% of patients, respectively (Table 1).

### 3.2. BP Control Status

Mean conventional BP was 140.3 ± 17.5/79.6 ± 10.7 mmHg. Mean total self-recorded BP was 131.5 ± 14.1/73.8 ± 7.6 mmHg. Morning and evening home BPs were 133.1 ± 16.5/75.8 ± 8.4 and 132.0 ± 15.7/73.5 ± 8.2 mmHg, respectively. Uncontrolled hypertension on conventional BP measurements was observed in 208 (56.7%) patients and on home-based BP measurements in 133 (36.2%) patients, according to clinic and home BP cutoff levels suggested in hypertension guidelines [8].

### 3.3. Morning-Evening Difference

The MEdiff ranged from −56.7 to 56.5 mmHg systolic (mean ± SD: 1.1 ± 12.5, *P* = 0.08) and −16.3 to 23.6 mmHg diastolic (mean ± SD: 2.3 ± 6.1, *P* < 0.0001) in self-recorded measurements.

In smokers, MEdiff was −6.39 ± 14.3 systolic (*P* < 0.01) and −0.89 diastolic (*P* = 0.4), being the home BP higher in the evening (134.5 ± 16.8/77.7 ± 7.0 mmHg) than in the morning (128.1 ± 17.1/76.8 ± 8.9).

After excluding smokers in the analysis, overall MEdiff reached statistical significance for both systolic (*P* < 0.01) and diastolic (*P* < 0.0001) BPs.

### 3.4. Determinants of Morning-Evening Difference

In univariate analyses, the variables analyzed were age, gender, ethnicity, smoking habit, office systolic and diastolic, BMI, diabetes, history of cardiovascular and cerebrovascular disease, number and class of antihypertensive drugs, and total cholesterol. Of these, the significantly predictive variables which were later incorporated to the multivariable linear regression model were age (*r* = 0.15, *P* < 0.01 systolic and *r* = 0.08, *P* < 0.001 diastolic), smoking (*r* = −8.5, *P* < 0.0001 systolic and *r* = −3.6, *P* < 0.0001 diastolic), total cholesterol (*r* = 0.7, *P* < 0.03 diastolic), and calcium-channel blocker use (*r* = 1.5, *P* < 0.02 diastolic).

In the multivariable analysis of our study, the independent determinants of elevated systolic MEdiff were age and smoking and of diastolic MEdiff were age, smoking, total cholesterol, and calcium-channel blockers. These determinants explained 6.2% and 8.5% of the variance in systolic and diastolic morning-evening home BP difference (Table 2).

## 4. Discussion

The purpose of our study was to evaluate the determinants of home-based MEdiff in Argentine patients who were referred to the Hypertension Section of our hospital to perform an HBPM. Our main finding in 367 subjects was that older age, smoking, total cholesterol, and use of calcium-channel blockers were independent determinants of the home-based MEdiff. To the best of our knowledge, these are the first data of the kind gathered from a South American group, mainly comprising Caucasian urban middle-class individuals, predominantly females.

### 4.1. Morning-Evening Home BP Profile

In the subjects of our study we found that, with the exception of smokers, in which home BP had a higher evening profile, home diastolic BP was significantly higher in the morning than in the evening and after excluding smokers from our cohort both systolic and diastolic BPs were significantly higher in the morning. These findings are similar to those studies performed in Northeast Asia [13, 15, 16, 25, 26] but different from those in Europe [14, 27, 28].

Ethnic variations have been explained before partly by the difference in evening BP measuring times: for instance, the Japanese guidelines [12] recommended measuring evening home BP before going to bed instead of using a fixed time, so that Asian measurements tended to be performed later than those in Western studies. Other factors related to these differences were linked to lifestyle habits that usually decrease BP, such as the Japanese custom of taking a nocturnal bath, which may lower the BP for at least 1 hour [37] and drinking an alcoholic beverage in the evening [16, 19, 25, 38].

In our study, patients were instructed to take the measurements before supper, in order to prevent a postprandial effect on evening BP measurements, which has already been characterized with HBPM after lunch [20] but may occur after supper being a reflection of the same pathophysiological process. Eating times in Argentina are usually between 8 and 11 p.m. In this respect, the time frame used by us is marginally later than the European but still reflects a more active period of the day than the Japanese.

Since taking a night-time shower or drinking alcohol are very uncommon in our setting, we would assume that there was not an association between evening BP decreases and these circumstances in our cohort of patients. However, since our results were more similar to the Japanese, it may be well expected that other factors may have played a role in decreasing evening home BP or increasing morning home BP other than the aforementioned lifestyle habits and the HBPM schedule.

One of the most likely relevant factors influencing the circadian home BP pattern in this study may have been the fact that all the subjects were under medication. The type, dosage, timing, and pharmacokinetic profiles of the antihypertensive drugs used may partly explain these BP differences, especially since subjects were instructed to measure morning home BP before the intake of medication. These drugs and time-related features may have also exaggerated the MEdiff in those studies which showed a similar morning-higher-than-evening pattern, such as the Japanese study of Ishikawa et al. [15] in treated patients, who found MEdiff mean values noticeably higher than ours (7.9 mmHg versus 1.1 mmHg, resp.), a fact that may be partly explained by distinct drug-related reasons, namely, evening-time medication.

Typically, medications are taken once a day, mostly in the morning, and the peak of the antihypertensive effect is observed in the evening. However, none of the studies has specified timing of medication nor associations with simple versus long-acting agents nor treatment scheduling.

### 4.2. Calcium-Channel Blockers (CCBs)

In our study, CCBs appeared as the only group of drugs to independently determine home-based diastolic MEdiff.

The evidence on the effect of antihypertensive drugs by group on self-measured MEdiff is very limited. In previous studies, Ikeda et al. [33] showed that the group with morning hypertension used higher doses of amlodipine compared to the controls and Kawabe et al. [25], in an urban population of Japanese hypertensives, found that the higher morning home BP was notable in patients who were taking antihypertensive drugs only in the morning, with CCBs being the drugs more often used (67%).

On the other hand, Ishikawa et al. [15], in a protocol based on average readings from 3 consecutive days, found an association only with *β*-blockers and the authors hypothesized that this finding was probably due to the effect upon the predominant *α*-sympathetic activation in the morning. Johansson et al. in the Finn-home study [14] found that use of antihypertensive medication was an independent predictor of MEdiff but did not provide an analysis by drug types.

In day-by-day home BP variability, the evidence is also rather limited and shows a favourable effect of CCBs (amlodipine, in particular) but not of *β*-blockers, which is consistent with data from office and ambulatory BP variability, implying that there are common mechanisms influencing home-based variability as for office and ambulatory BP variability [39].

Our data are in contradiction with these favourable effects of CCBs on home BP variability, which have been attributed to many intrinsic features such as their vasodilating effects on peripheral muscular arteries, decreased peripheral resistance, increased baroreflex sensitivity, reduced arterial stiffness, and long elimination half-lives. They also contradict the results with *β*-blockers and the *α*-sympathetic activation hypothesis. Since only one study [25] could establish an association regarding timing and none of the studies provided thorough data regarding pharmacokinetic characteristics of the medication and duration of therapy, we could only conjecture that night-time medication, which is relatively common in our setting, may have played a role in the results, but no further conclusions could be drawn until further data are collected.

### 4.3. Total Cholesterol

To the best of our knowledge, this is the first study to show an association between total cholesterol and home-based diastolic MEdiff. Lee et al. [26] previously showed a higher prevalence of metabolic syndrome in patients with morning hypertension compared to controls (59.5% versus 49.5%, *P* = 0.019), but when they analyzed the separate components of metabolic syndrome, dyslipidemia was not significantly different between patients with morning hypertension and the controls.

However, total cholesterol has been linked to other forms of variability such as within visit BP variability [40] and diurnal/nocturnal short term BP variability evaluated through ABPM in patients with CKD [41].

### 4.4. Age

Older age was found to be a determinant for both systolic and diastolic MEdiff levels in this study. This is consistent with findings by other authors [15, 26] and may reflect key physiological mechanisms involved with BP variability such as increased arterial stiffness and autonomic failure due to impaired sympathetic baroreflex sensitivity, which impedes the counter response of the *α*-adrenergic activation, especially in the morning [42]. The BP rise in the early hours is dependent on *α*-adrenergic activity and has been associated with silent cerebrovascular disease in the elderly. Therefore, detection of morning-evening differences through self-measured BP may be helpful in preventing target organ damage in this special population.

### 4.5. Smoking

In our study, smoking was an independent determinant of both systolic and diastolic home MEdiff levels and was associated with a decrease in MEdiff levels. Home systolic BP in this group was significantly higher in the evening than in the morning.

In this respect, information from previous studies is consistent with these results. The J-MORE study [15] found that there was a reduced risk for exaggerated MEdiff in smokers, and the smoking prevalence tended to be lower in the highest MEdiff quartile. In the Finn-Home study [14], the findings suggested that daytime smoking may elevate evening home BP and consequently reduce MEdiff values.

Tobacco consumption has interindividual behavioural variations during the day because each person has different sleeping rhythms and daytime customs when it comes to lighting a cigarette. However, the evening-type smokers of both genders are more likely to be current and ever smokers due to nicotine dependence than the morning types [43].

Pathophysiologically, the effect may be partly explained by several mechanisms such as increased arterial stiffness and aortic wave reflection [44] and an exaggerated daytime pressor response due to sympathetic activation produced during cigarette smoking and the partial inability to reflexly counteract the adrenergic effect because of baroreflex impairment [45].

### 4.6. Study Limitations

Due to the hospital-based nature of our study, it is difficult to generalize the present findings to the overall community. In addition, the patients affiliated to our health plan are mainly urban Argentine middle-class individuals of European descent (in the majority of cases Italian and Spanish) who may not reflect other ethnicities living in South America.

Since the information on smoking habits and intake of medications was obtained from an electronic medical registry, the number of smokers and drugs used by the patients may have been underestimated. In addition, timing of smoking and intake of medication were not controlled.

Interpretation of the results should be analyzed carefully due to the cross-sectional nature of the study which precludes cause-effect relationships and also because an exaggerated response in home MEdiff may be a reflection of two separate phenomena, an increase in morning BP and/or a decrease in evening BP.

In a general population, Asayama et al. [46] showed that new indices of home variability such as variability independent of the mean, the difference between maximum and minimum BP, and average real variability do not substantially refine risk profiling over and beyond the BP level. The added value of home-based MEdiff in risk stratification has not been characterized yet. Further studies will be necessary to evaluate the clinical significance of this parameter and determine which method of home BP variability is the most reliable one in cardiovascular risk prediction.

In conclusion, in a cohort of Argentine medicated patients, in whom timing and dosage of treatment were not controlled, older age, smoking, total cholesterol, and use of calcium-channel blockers were independent determinants of home-based MEdiff. BP was higher in the morning except for smokers.

## Figures and Tables

**Table 1:** Characteristics of the study population.

Characteristic	Total
Number of subjects (%)	367
Women	242 (65.9)
Caucasians	365 (99.5)
Smokers	43 (11.7)
Uncontrolled hypertension	133 (36.2)
Diabetes mellitus	39 (10.6)
Previous cardiovascular disease	42 (11.4)
Previous cerebrovascular disease	24 (6.5)
Mean (±SD) characteristic	
Age, y	66.2 (14.5)
Body mass index, kg/m^2^	28.1 (4.5)
Conventional BP, mmHg	
Systolic	140.3 (17.5)
Diastolic	79.6 (10.7)
Self-recorded systolic BP, mmHg	
All measurements	131.5 (14.1)
Morning	133.1 (16.5)
Evening	132.0 (15.7)
Morning-evening difference	1.1 (12.5)
Self-recorded diastolic BP, mmHg	
All measurements	73.8 (7.6)
Morning	75.8 (8.4)
Evening	73.5 (8.2)
Morning-evening difference	2.3 (6.1)
Self-recorded pulse rate, beats/min	
All measurements	71.3 (10.9)
Morning	69.4 (11.2)
Evening	72.0 (11.6)
Number of self-recorded BP measurements	24.7 (2.9)
Number of antihypertensive drugs	2.2 (1.0)
Serum cholesterol, mmol/L	4.89 (1.0)

Uncontrolled hypertension was a home blood pressure of at least 135 mmHg systolic or 85 mmHg diastolic. Diabetes mellitus was a self-reported diagnosis, a fasting or random blood glucose level of 7.0 mmol/L (126 mg/dL) or 11.1 mmol/L (200 mg/dL) or higher, or a use of antidiabetic drugs. Smoking was daily use of tobacco products. Previous cardiovascular disease included ischemic heart disease, atrial fibrillation, or congestive heart failure. Previous cerebrovascular disease included transient ischemic attack or stroke.

**Table 2:** Multivariable linear regression model for morning-evening blood pressure difference.

Variable	Beta-coefficient (95% CI)	*P* value
Systolic morning-evening home BP difference		
Age	0.12 (0.03–0.21)	0.007
Smoking habit	−7.52 (−11.44–[−3.61])	<0.0001
Diastolic morning-evening home BP difference		
Age	0.07 (0.03–0.12)	0.001
Total cholesterol	0.99 (0.38–1.6)	0.002
CCB use	1.44 (0.21–2.66)	0.02
Smoking habit	−2.91 (−4.81–[−1.02])	0.003

Adjusted *R* ^2^ values for the model were 6.2% for systolic and 8.5% (*P* < 0.0001) for diastolic morning-evening BP difference, respectively.

## References

[1] Rickerby J., Woodward J. (2003). Patients' experiences and opinions of home blood pressure measurement. *Journal of Human Hypertension*.

[2] Staessen J. A., Thijs L., Ohkubo T., Kikuya M., Richart T., Boggia J., Adiyaman A., Dechering D. G., Kuznetsova T., Thien T., De Leeuw P., Imai Y., O'Brien E., Parati G. (2008). Thirty years of research on diagnostic and therapeutic thresholds for the self-measured blood pressure at home. *Blood Pressure Monitoring*.

[3] Asayama K., Thijs L., Brguljan-Hitij J., Niiranen T. J., Hozawa A., Boggia J., Aparicio L. S., Hara A., Johansson J. K., Ohkubo T., Tzourio C., Stergiou G. S., Sandoya E., Tsuji I., Jula A. M., Imai Y., Staessen J. A., Kikuya M., Inoue R., Satoh M., Hosaka M., Utsugi M. T., Hirose T., Fukushima N., Obara T., Metoki H., Reunanen A., Ohmori-Matsuda K., Kuriyama S., Kakizaki M., Kollias A., Thomopoulou G., Kalogeropoulos P., Skeva I., Nasothimiou E., Pantazis N., Baibas N., Mountokalakis T., Cauwenberghs N., Zhang Z., Wei F., Knez J., Odili A., Gu Y., Liu Y., Jin Y., Jacobs L., Kuznetzova T. (2014). Risk stratification by self-measured home blood pressure across categories of conventional blood pressure: a participant-level meta-analysis. *PLoS Medicine*.

[4] Sakuma M., Imai Y., Nagai K., Watanabe N., Sakuma H., Minami N., Satoh H., Abe K. (1997). Reproducibility of home blood pressure measurements over a 1-year period. *The American Journal of Hypertension*.

[5] Ohkubo T., Imai Y., Tsuji I., Nagai K., Kato J., Kikuchi N., Nishiyama A., Aihara A., Sekino M., Kikuya M., Ito S., Satoh H., Hisamichi S. (1998). Home blood pressure measurement has a stronger predictive power for mortality than does screening blood pressure measurement: a population-based observation in Ohasama, Japan. *Journal of Hypertension*.

[6] Niiranen T. J., Hänninen M.-R., Johansson J., Reunanen A., Jula A. M. (2010). Home-measured blood pressure is a stronger predictor of cardiovascular risk than office blood pressure: the finn-home study. *Hypertension*.

[7] Bobrie G., Chatellier G., Genes N., Clerson P., Vaur L., Vaisse B., Menard J., Mallion J.-M. (2004). Cardiovascular prognosis of “masked hypertension” detected by blood pressure self-measurement in elderly treated hypertensive patients. *Journal of the American Medical Association*.

[8] Mancia G., Fagard R., Narkiewicz K. (2013). 2013 ESH/ESC guidelines for the management of arterial hypertension: the Task Force for the Management of Arterial Hypertension of the European Society of Hypertension (ESH) and of the European Society of Cardiology (ESC). *European Heart Journal*.

[9] Parati G., Stergiou G. S., Asmar R., Bilo G., De Leeuw P., Imai Y., Kario K., Lurbe E., Manolis A., Mengden T., O'brien E., Ohkubo T., Padfield P., Palatini P., Pickering T., Redon J., Revera M., Ruilope L. M., Shennan A., Staessen J. A., Tisler A., Waeber B., Zanchetti A., Mancia G. (2008). European society of hypertension guidelines for blood pressure monitoring at home: a summary report of the second international consensus conference on home blood pressure monitoring. *Journal of Hypertension*.

[10] NICE (2011). *Hypertension: Clinical Management of Primary Hypertension in Adults (NICE Clinical Guideline 127)*.

[11] Chobanian A. V., Bakris G. L., Black H. R. (2003). The seventh report of the joint National committee on prevention, detection, evaluation, and treatment of high blood pressure: the JNC 7 report. *The Journal of the American Medical Association*.

[12] Imai Y., Kario K., Shimada K., Kawano Y., Hasebe N., Matsuura H., Tsuchihashi T., Ohkubo T., Kuwajima I., Miyakawa M. (2012). The japanese society of hypertension guidelines for self-monitoring of blood pressure at home (Second Edition). *Hypertension Research*.

[13] Imai Y., Nishiyama A., Sekino M. (1999). Characteristics of blood pressure measured at home in the morning and in the evening: the Ohasama Study. *Journal of Hypertension*.

[14] Johansson J. K., Niiranen T. J., Puukka P. J., Jula A. M. (2011). Factors affecting the difference between morning and evening home blood pressure: the Finn-Home study. *Blood Pressure*.

[15] Ishikawa J., Kario K., Hoshide S., Eguchi K., Morinari M., Kaneda R., Umeda Y., Ishikawa S., Kuroda T., Hojo Y., Shimada K. (2005). Determinants of exaggerated difference in morning and evening blood pressure measured by self-measured blood pressure monitoring in medicated hypertensive patients: jichi Morning Hypertension Research (J-MORE) study. *The American Journal of Hypertension*.

[16] Ito K., Obara T., Ohkubo T., Gonokami K., Shinki T., Shibamiya T., Nakashita M., Kobayashi M., Funahashi J., Hara A., Metoki H., Asayama K., Inoue R., Kikuya M., Mano N., Imai Y. (2009). Influence of home blood pressure measuring conditions in the evening on the morning-evening home blood pressure difference in treated hypertensive patients: the J-HOME study. *Blood Pressure Monitoring*.

[17] Lavie-Nevo K., Pillar G. (2006). Evening-morning differences in blood pressure in sleep apnea syndrome: effect of gender. *American Journal of Hypertension*.

[18] Johansson J. K., Kronholm E., Jula A. M. (2011). Variability in home-measured blood pressure and heart rate: associations with self-reported insomnia and sleep duration. *Journal of Hypertension*.

[19] Kawabe H., Saito I. (2009). Determinants of exaggerated difference in morning and evening home blood pressure in Japanese normotensives. *Hypertension Research*.

[20] Barochiner J., Alfie J., Aparicio L. S., Cuffaro P. E., Rada M. A., Morales M. S., Galarza C. R., Marín M. J., Waisman G. D. (2014). Postprandial hypotension detected through home blood pressure monitoring: a frequent phenomenon in elderly hypertensive patients. *Hypertension Research*.

[21] Hashimoto T., Kikuya M., Ohkubo T. (2012). Home blood pressure level, blood pressure variability, smoking, and stroke risk in Japanese men: the ohasama study. *American Journal of Hypertension*.

[22] Seki M., Inoue R., Ohkubo T., Kikuya M., Hara A., Metoki H., Hirose T., Tsubota-Utsugi M., Asayama K., Kanno A., Obara T., Hoshi H., Totsune K., Satoh H., Imai Y. (2010). Association of environmental tobacco smoke exposure with elevated home blood pressure in Japanese women: the Ohasama study. *Journal of Hypertension*.

[23] Hoshide S., Kario K., Yano Y. (2014). Association of morning and evening blood pressure at home with asymptomatic organ damage in the J-HOP study. *The American Journal of Hypertension*.

[24] Okada T., Nakao T., Matsumoto H., Nagaoka Y. (2008). Value of morning home blood pressure as a predictor of decline in renal function in patients with chronic kidney disease. *American Journal of Nephrology*.

[25] Kawabe H., Saito I., Saruta T. (2005). Status of home blood pressure measured in morning and evening: evaluation in normotensives and hypertensives in Japanese urban population. *Hypertension Research*.

[26] Lee J. H., Bae J. W., Park J. B., Park C. G., Youn H. J., Choi D. J., Ahn Y. K., Shin J. H., Rim S. J., Bae J. H., Kim D. W. (2011). Morning hypertension in treated hypertensives: baseline characteristics and clinical implications. *Korean Circulation Journal*.

[27] Den Hond E., Celis H., Fagard R., Keary L., Leeman M., O'Brien E., Vandenhoven G., Staessen J. A. (2003). Self-measured versus ambulatory blood pressure in the diagnosis of hypertension. *Journal of Hypertension*.

[28] De Gaudemaris R., Chau N. P., Mallion J.-M. (1994). Home blood pressure: variability, comparison with office readings and proposal for reference values. *Journal of Hypertension*.

[29] Stergiou G. S., Nasothimiou E. G., Kalogeropoulos P. G., Pantazis N., Baibas N. M. (2010). The optimal home blood pressure monitoring schedule based on the Didima outcome study. *Journal of Human Hypertension*.

[30] Asayama K., Ohkubo T., Kikuya M., Obara T., Metoki H., Inoue R., Hara A., Hirose T., Hoshi H., Hashimoto J., Totsune K., Satoh H., Imai Y. (2006). Prediction of stroke by home “morning” versus “evening” blood pressure values: the Ohasama study. *Hypertension*.

[31] Asayama K., Ohkubo T., Hara A. (2009). Repeated evening home blood pressure measurement improves prognostic significance for stroke: a 12-year follow-up of the ohasama study. *Blood Pressure Monitoring*.

[32] Shibuya Y., Ikeda T., Gomi T. (2007). Morning rise of blood pressure assessed by home blood pressure monitoring is associated with left ventricular hypertrophy in hypertensive patients receiving long-term antihypertensive medication. *Hypertension Research*.

[33] Ikeda T., Gomi T., Shibuya Y., Matsuo K., Kosugi T., Oku N., Uetake Y., Kinugasa S., Furutera R. (2004). Morning rise in blood pressure is a predictor of left ventricular hypertrophy in treated hypertensive patients. *Hypertension Research*.

[34] Matsui Y., Eguchi K., Shibasaki S. (2009). Association between the morning-evening difference in home blood pressure and cardiac damage in untreated hypertensive patients. *Journal of Hypertension*.

[35] Barochiner J., Cuffaro P. E., Aparicio L. S., Elizondo C. M., Giunta D. H., Rada M. A., Morales M. S., Alfie J., Galarza C. R., Waisman G. D. (2011). Reproducibility and reliability of a 4-day HBPM protocol with and without first day measurements. *Revista de la Facultad de Ciencias Médicas*.

[36] Artigao L. M., Llavador J. J., Puras A., López Abril J., Rubio M. M., Torres C., Vidal A., Sanchis C., Divisón J. A., Naharro F., Caldevilla D., Fuentes G. (2000). Evaluation and validation of Omron Hem 705 CP and Hem 706/711 monitors for self-measurement of blood pressure. *Atencion Primaria*.

[37] Kawabe H., Saito I. (2006). Influence of nighttime bathing on evening home blood pressure measurements: how long should the interval be after bathing?. *Hypertension Research*.

[38] Kawabe H., Saito I., Saruta T. (2007). Effects of nighttime alcohol intake on evening and next morning home blood pressure in Japanese normotensives. *Clinical and Experimental Hypertension*.

[39] Stergiou G. S., Ntineri A., Kollias A., Ohkubo T., Imai Y., Parati G. (2014). Blood pressure variability assessed by home measurements: a systematic review. *Hypertension Research*.

[40] Shin J. H., Kim B. K., Lim Y.-H. (2013). Within-visit blood pressure variability: relevant factors in the general population. *Journal of Human Hypertension*.

[41] Sakai M., Tamura K., Tanaka Y., Tsurumi Y., Okano Y., Koide Y., Endoh T., Matsushita K., Kihara M., Hirawa N., Toya Y., Tokita Y., Ohnishi T., Umemura S. (2005). Analysis of factors that affect short-term blood pressure variability in patients with chronic renal failure. *Clinical and Experimental Hypertension*.

[42] Okada Y., Galbreath M. M., Shibata S. (2013). Morning blood pressure surge is associated with arterial stiffness and sympathetic baroreflex sensitivity in hypertensive seniors. *American Journal of Physiology—Heart and Circulatory Physiology*.

[43] Broms U., Kaprio J., Hublin C., Partinen M., Madden P. A. F., Koskenvuo M. (2011). Evening types are more often current smokers and nicotine-dependent-a study of Finnish adult twins. *Addiction*.

[44] Jatoi N. A., Jerrard-Dunne P., Feely J., Mahmud A. (2007). Impact of smoking and smoking cessation on arterial stiffness and aortic wave reflection in hypertension. *Hypertension*.

[45] Grassi G., Seravalle G., Calhoun D. A., Bolla G. B., Giannattasio C., Marabini M., Del Bo A., Mancia G. (1994). Mechanisms responsible for sympathetic activation by cigarette smoking in humans. *Circulation*.

[46] Asayama K., Kikuya M., Schutte R., Thijs L., Hosaka M., Satoh M., Hara A., Obara T., Inoue R., Metoki H., Hirose T., Ohkubo T., Staessen J. A., Imai Y. (2013). Home blood pressure variability as cardiovascular risk factor in the population of ohasama. *Hypertension*.

